# Transcription Factor *VvbHLH137* Positively Regulates Anthocyanin Accumulation in Grape (*Vitis vinifera*)

**DOI:** 10.3390/plants14060871

**Published:** 2025-03-11

**Authors:** Zaozhu Niu, Zhichao Zhang, Yanzhuo Zhao, Lifeng Xuan, Zhan Chen, Lili Yang

**Affiliations:** Shijiazhuang Institute of Fruit Trees, Hebei Academy of Agriculture and Forestry Sciences, Shijiazhuang 050061, China; niuzaozhu0311@163.com (Z.N.); zhangzc927@163.com (Z.Z.); zyz432@126.com (Y.Z.); znlxlf@163.com (L.X.)

**Keywords:** grape, anthocyanin synthesis, RNA-seq, bHLH transcription factors, *VvDFR*, *VvF3H*

## Abstract

Grape (*Vitis vinifera*) is a popular fruit with a rich color, favorable taste, and high nutritional quality. The formation of the color of its berries is primarily determined by anthocyanin composition and concentration. Basic helix–loop–helix proteins (bHLHs) serve as critical modulators of anthocyanin synthesis, yet many bHLHs in grape have not been systematically studied and remain uncharacterized. In this study, we tracked and detected berry components in ‘Moldova’ grapes during three developmental stages using UPLC-MS/MS and identified malvidin derivatives as the primary main anthocyanins. Our transcriptome sequencing analysis revealed 40 genes and several transcription factors (TFs) involved in anthocyanin pathways and berry coloration, with *VvCHS2* (*Vitvi05g01044*) showing the highest expression. Among TFs, six bHLH candidates were identified, and *VvbHLH137* was determined to positively regulate anthocyanin synthesis. The over-expression of *VvbHLH137* in Arabidopsis thaliana significantly augmented the anthocyanin content. In addition, *VvbHLH137* was found to form interactions with *VvMYB15*, *VvMYB44*, and *VvMYB306* to impact anthocyanin accumulation. It also directedly stimulates *VvDFR* and *VvF3H* transcription via binding to their promoters. These findings provide insights into anthocyanin synthesis in grapes and support molecular breeding efforts for grape cultivars with enhanced coloration.

## 1. Introduction

Grape (*Vitis vinifera*) is a world-renowned fruit consumed fresh and used for raisin production and winemaking [1]. The coloration of its berries, a key quality attribute, is determined by the anthocyanin accumulation in the peel. At present, with the frequency of extreme weather and the popularization of facility cultivation, grape coloring has become a major production problem. As everyone knows, six primary anthocyanin types (cyanidin, peonidin, pelargonidin, delphinidin, malvidin, petunidin, and their derivatives) contribute to the diversity of grape skin colors based on their combinations and concentrations [2]. Anthocyanin synthesis, part of the flavonoid pathway within the phenylpropanoid system, has been thoroughly studied in fruit crops [3]. Genes encoding essential enzymes for its synthesis, including phenylalanine ammonialyase (PAL), flavanone-3-hydroxylase (F3H), chalcone synthase (CHS), chalcone isomerase (CHI), flavonoid 3′-monooxygenase (F3′H), flavonoid 3′5′-monooxygenase (F3′5′H), anthocyanidin synthase (ANS), and UDP-glucoside: flavonoid-3-O-glucosyltransferase (UFGT), have been identified [4,5,6,7]. The regulation of anthocyanin accumulation involves transcription factors (TFs) from three main categories: bHLH, MYB, and WD40, which are often assembled in the MBW complex to function [8,9,10,11]. MYB TFs are well-studied regulators for anthocyanin synthesis. For instance, *VvMYBA1* and *VvMYBA2* control *UFGT* expression to regulate grape coloration. Functional loss mutations in these MYB genes block anthocyanin accumulation, resulting in green grapes. These mutations include retrotransposon insertion in *VvMYBA1* and coding region alterations in *VvMYBA2* [12,13].

bHLH TFs, the second largest plant TF family, have a conserved domain (~60 amino acids) with a basic DNA-binding region and a helix–loop–helix at the C terminus [14,15]. These TFs regulate anthocyanin synthesis themselves or by interacting with MYB proteins and binding to structural gene promoters [16,17,18]. The first bHLH TF, *R1*, discovered in maize (*Zea mays*) by Ludwig and Wessler, regulates anthocyanin synthesis by forming the MYB-bHLH-WD40 complex via interacting with the R2R3-MYB protein and WD40 complex [19]. bHLH TFs, such as *TT8*, *MdbHLH3 (Malus domestica)* [20,21], *AcbHLH42 (Actinidia Chinensis)* [22], *PsbHLH1 (Paeonia Suffruticosa)* [23], and *CsbHLH89 (Camellia Sinensis)* [24], positively regulate anthocyanin synthesis in fruits, seeds, or petals. In apples, *MdbHLH3* and *MdbHLH33* are associated with *MdMYB10* to enhance *MdDFR* and *MdUFGT* promoter activity, augmenting anthocyanin synthesis [20]. Similarly, peach *PpbHLH3 (Prunus Persica)* is essential for *PpMYB10* to activate *PpDFR* and *PpUFGT* promoters [25]. In strawberries (*Fragaria Vesca*), *FvMYB10* only enhances the levels of *FvDFR* and *FvUFGT*, essential genes for phytochrome production [26]. Bayberry (*Myrica Rubra*) *MrbHLH1* interacts with *MrMYB1* to form a transcriptional complex that stimulates structural genes and drives anthocyanin accumulation. In contrast, its homolog *MrbHLH2* lacks this function [27]. In onion *(Allium Cepa)*, *AcB2* interacts with *AcMYB1* to modulate its direct interaction with the promoters of *AcANS* and *AcF3H1*, which encode anthocyanin synthase and flavonoid 3-hydroxylase 1, respectively, thereby boosting their levels [28]. However, some bHLH TFs negatively regulate anthocyanin synthesis, such as *CpbHLH1 (Chimonanthus Praecox)* [29], *BnbHLH92a (Brassica Napus)* [30], and *LcbHLH92 (Leymus chinensis)* [31]. For instance, the over-expression of *CpbHLH1* from wintersweet dramatically attenuates anthocyanin in transgenic tobacco and Arabidopsis plants, making it the first dicot bHLH TF identified to suppress anthocyanin synthesis [29]. Similarly, the over-expression of *BnbHLH92a* (*Brassica napus*) in Arabidopsis and rapeseed reduces anthocyanin levels by physically interacting with *BnTTG1* and repressing *BnTT18* promoter activity [30]. In grapes, bHLH TFs play critical roles in anthocyanin accumulation. For example, *VvMYC1* interacts with *VvMYB5a/VvMYB5b*, *VvMYBA1/A2*, and *VvMYBPA1*, enhancing *CHI*, *UFGT*, and *ANR* expression levels in the downstream pathway, thereby increasing anthocyanin synthesis [32]. Similarly, *VvbHLH1* over-expression in transgenic Arabidopsis overrepresented anthocyanin biosynthetic genes [33]. However, the precise roles of many bHLH TFs in grape anthocyanin accumulation remain undefined and require further exploration.

The ‘Moldova’ grape variety is a valuable variety and breeding material due to its high resistance to downy mildew, attractive coloring, high yield, late ripening, and storage resistance. This study aimed to explore the primary anthocyanin types and essential genes responsible for grape coloration. Using UPLC-MS/MS and transcriptome analyses at three berry developmental stages, we identified *VvbHLH137* as a pivotal regulator of anthocyanin accumulation. We further elucidated its underlying molecular mechanism, providing insight into grape color variation and laying a foundation for molecular breeding to develop grapes with desirable coloration.

## 2. Results

### 2.1. Changes in Anthocyanin Composition Drive Grape Coloration During Development

**Figure 1:**
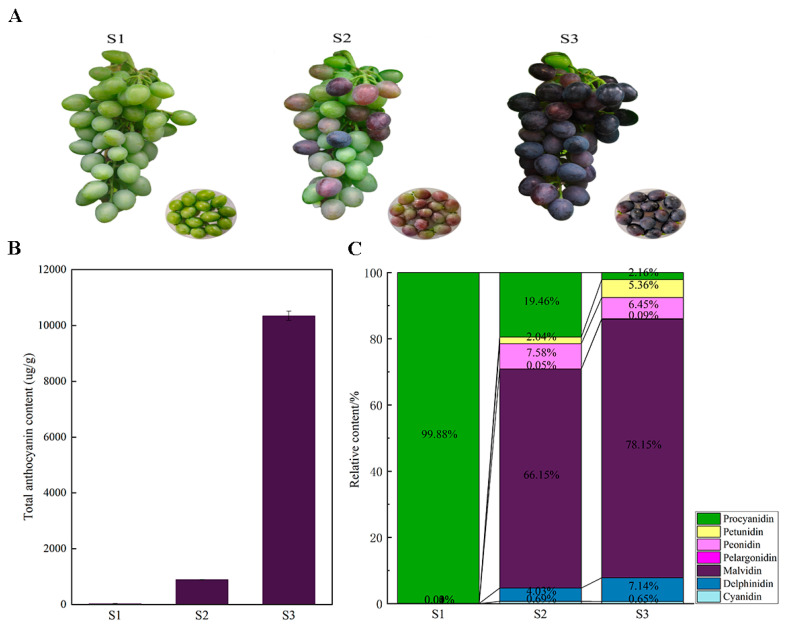
Changes in color and anthocyanin metabolites during grape development. (**A**) Visual representation of grape berries at three developmental stages: S1 (51 days post-bloom), S2 (65 days post-bloom), and S3 (79 days post-bloom). (**B**) Total anthocyanin content (μg/g) in grape peels across stages, presented as means ± SD (3 replicates). (**C**) Distribution of different anthocyanin metabolites at different stages.

To investigate changes in anthocyanin composition and grape coloration, Moldova grape berries were collected at 51 (S1), 65 (S2), and 79 (S3) days after full bloom. The berry color transitioned from green (S1) to purple-red (S2) and finally to purple-black (S3) (Figure 1A). A quantitative analysis showed a continuous increase in total anthocyanin content during berry development from 31.46 μg/g (S1) to 10,345.66 μg/g (S3) (Figure 1B).

A UPLC-MS/MS analysis of anthocyanin metabolites identified 51 distinct compounds, including eleven malvidin derivatives, ten delphinidin derivatives, nine peonidin derivatives, eight cyanidin derivatives, five procyanidin derivatives, five petunidin derivatives, and three pelargonidin derivatives (Figure 1C, Table S1). The number and content of anthocyanin metabolites varied across the growth stages: 18 were detected at S1, 35 at S2, and 48 at S3. At S1, procyanidins (B1, B2, B3, B4, and C1) dominated (98.92% of the total anthocyanins). As the berries matured, other anthocyanins gradually accumulated, with malvidin derivatives becoming the most abundant at S3, followed by delphinidin derivatives. Among them, malvidin-3,5-O-diglucoside showed the highest increase, from undetectable levels to 7726.99 μg/g, becoming the predominant abundant compound, malvidin-3-O-glucoside was the second most abundant (2259.61 μg/g), and malvidin-3-O-(6″-O-caffeoyl)-glucoside was the least abundant (2.50 μg/g) (Table S1). These results confirm that the dynamic changes in anthocyanin metabolites are key to grape peel coloration.

### 2.2. Transcriptome Profiling of Grape Berries During Development

To uncover the mechanisms for anthocyanin synthesis, RNA sequencing was performed on grape peels at three developmental stages in triplicate. Nine cDNA libraries were established. After filtering out adaptors and poor-quality reads, over 89% of the clean reads were matched to the grape reference genome (Vitis_vinifera.PN40024.v4.dna.toplevel.fa.gz). Approximately 60.75 gigabytes (Gb) of clean data were produced, with a mean about 6.75 Gb per sample. The GC contents (%) of the nine libraries were from 45.32% to 47.07%, and the Q30 proportion was at least 92.75% (Table S2). The mapping rates were 93.28% to 94.48%, with unique mapping rates between 89.82% and 91.03% (Table S2). These findings demonstrate that the sequencing results were of sufficient quality and accuracy for a deeper investigation.

### 2.3. Exploration of Differentially Expressed Genes (DEGs)

**Figure 2:**
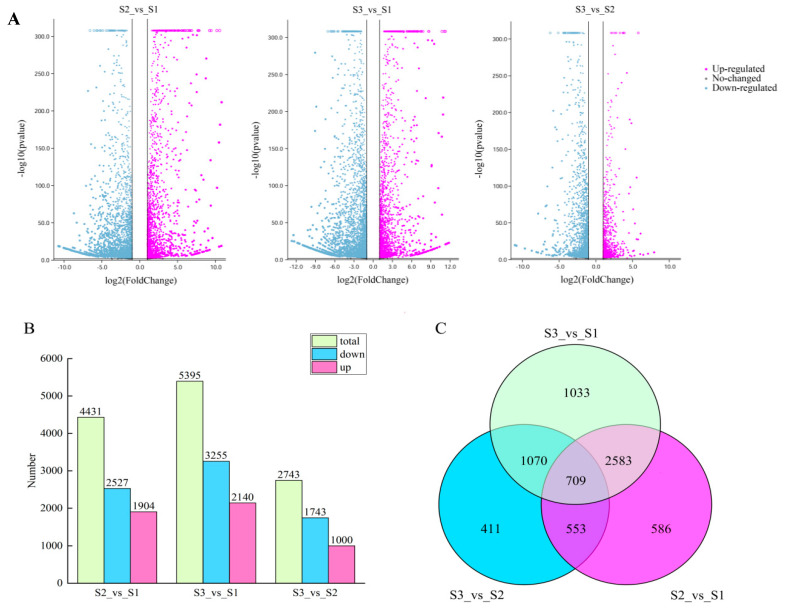
DEGs over the three developmental stages. (**A**) Volcano plots illustrating DEGs. (**B**) Bar graph showing the total numbers of DEGs. (**C**) Venn diagram displaying the overlap of DEGs.

Based on a threshold of |log2Fold Change| ≥ 1 and an FDR < 0.05, 6945 DEGs were uncovered, including 1904 overrepresented and 2527 underrepresented DEGs between S2 and S1, 3255 underrepresented and 2140 overrepresented DEGs between S3 and S1, and 1000 overrepresented and 1743 underrepresented DEGs between S3 and S2 (Figure 2A,B). The Venn diagram revealed 586, 1033, and 411 unique DEGs for the comparisons of S2 vs. S1, S3 vs. S1, and S3 vs. S2, respectively, with 709 DEGs shared among all three groups (Figure 2C). S3 vs. S1 exhibited more DEGs than S2 vs. S1, indicating increased gene involvement in coloration during later stages. Conversely, this number for S3 vs. S2 decreased, suggesting a persistent or suppressed expression of certain genes.

To further understand DEG functions, GO and KEGG analyses were applied (Figures S1 and S2). These DEGs were concentrated in 41 GO terms, covering 22 biological processes, two cellular components, and 16 molecular functions. Among the biological processes, “metabolic process (GO:0008152)”, “cellular process (GO:0009987)”, “response to stimulus (GO:0050896)”, “biological regulation (GO:0065007)”, and “regulation of biological process (GO:0050789)” were the primary enriched terms. Among the cellular process, “cellular anatomical entity (GO:0110165)” and “protein-containing complex (GO:0032991)” were the primary terms, with a predominance of the former. Among the molecular functions, “catalytic activity (GO:0003824)” and “binding (GO:0005488)” were the most enriched, followed by “transporter activity (GO:0005215)”, “transcription regulator activity (GO:0140110)”, and “ATP-dependent activity (GO:0140657)”.

The KEGG pathway analysis revealed 1410 DEGs enriched in 133 pathways for the S2 vs. S1 group, 1617 DEGs in 137 pathways for the S3 vs. S1 group, and 845 DEGs in 127 pathways for the S3 vs. S2 group. The key enriched pathways across the comparisons involved secondary metabolite biosynthesis (ko01110), plant hormone signal transduction (ko04075), metabolic pathways (ko01100), and plant–pathogen interaction (ko04626).

### 2.4. Identification of DEGs Responsible for Anthocyanin Synthesis

**Figure 3:**
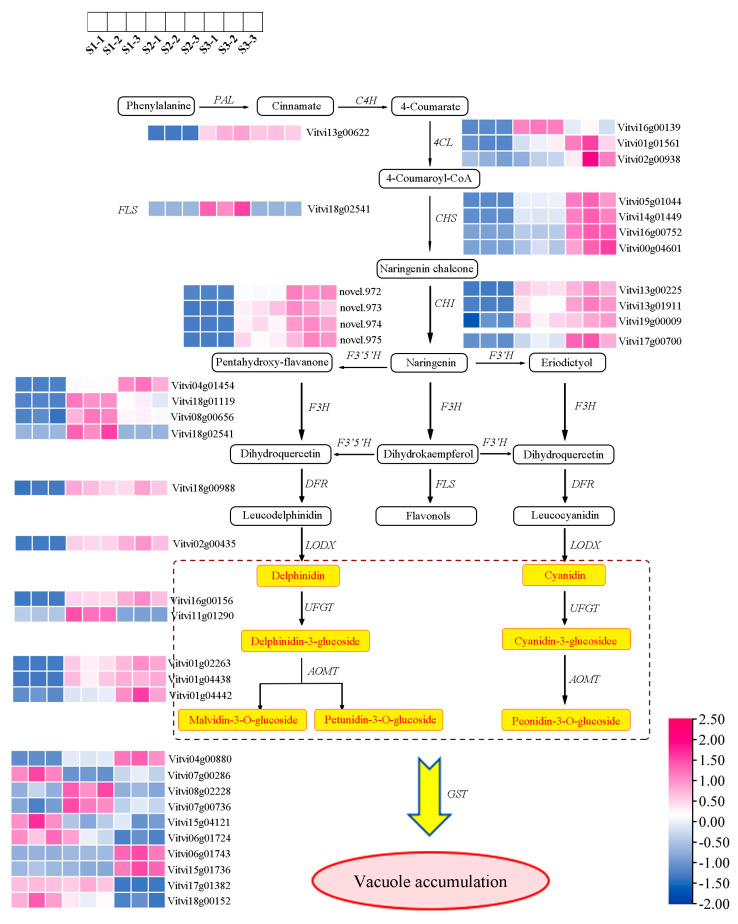
A simplified anthocyanin synthesis pathway with key DEG levels in grape peels. Each colored cell displays the average log2(FPKM), in which rose red and blue signify elevated and attenuated abundance, respectively, as shown by the color scale.

To investigate differences in anthocyanin production during development from S1 to S3, 40 anthocyanin-synthesis-related structural genes were identified as DEGs (Figure 3). Several genes showed differential expression at the early stages of anthocyanin synthesis, including one *PAL*, three *4CL*, three *CYP73A*, four *CHS*, and three *CHI* genes. Notably, *PAL (Vitvi13g00622)* had a lower level of expression at S1, with significantly higher levels at S2 and S3. Similarly, *CHS (Vitvi05g01044)* had a low level of expression at S1 but rapidly increased at S2 and S3, becoming the most highly expressed gene. The conversion of naringin to dihydrokaempferol, a critical step in anthocyanin synthesis, is catalyzed by *F3H* genes. Among these, *F3H (Vitvi04g01454)* showed a low expression level at S1, with a gradual increase with the fruit’s development. In contrast, other *F3H* genes (*Vitvi18g01119*, *Vitvi08g00656*, and *Vitvi18g02541*) were minimally expressed at S1, increased at S2, and decreased slightly at S3. F3′H and F3′5′H are fundamental for shaping the composition of anthocyanin precursors. *F3′H (Vitvi17g00700)* expression at S3 was nearly doubled that of S2 and triple that of S1. The four *F3′5′H* genes exhibited negligible expression levels at S1, with their expression levels gradually increasing as the fruit developed.

The late stages of anthocyanin synthesis are also crucial for anthocyanin accumulation. The *DFR* and *LODX* genes had lower expression levels at S1, but their expression levels gradually increased as the fruit developed. One of the two *UFGT* genes, *UFGT (Vitvi16g00156),* was upregulated, and the other, *UFGT (Vitvi11g01290),* was first increased and then decreased from the S1 to S3 stages. Three *AOMT* genes *(Vitvi01g02263*, *Vitvi01g04438*, and *Vitvi01g04442)* exhibited a steady increase in expression levels from S1 to S3. Among the 10 *GST* genes analyzed that had different expression patterns, two *(Vitvi04g00880* and *Vitvi06g01743)* showed continuous upregulation throughout the developmental stages, and *Vitvi04g00880* had the highest expression level, while the remaining genes exhibited special expression patterns.

### 2.5. Identification of TFs Linked to Anthocyanin Synthesis

**Figure 4:**
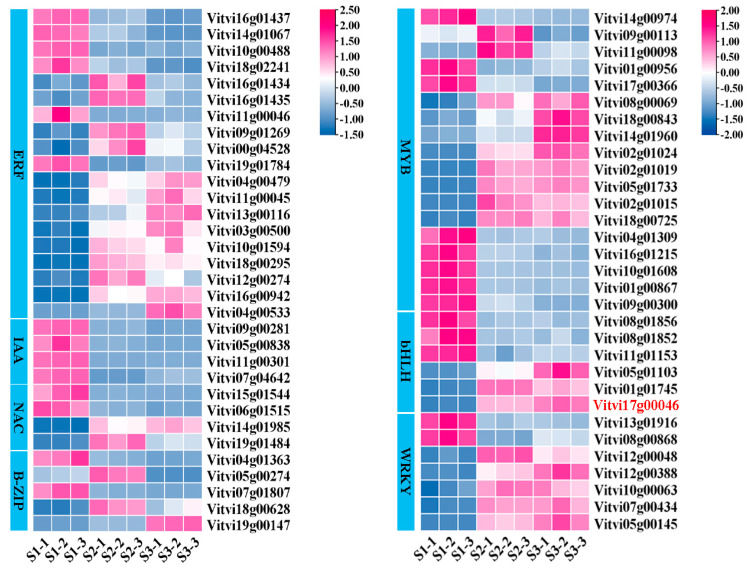
Expression of anthocyanin-synthesis-related TFs in grape. Each colored cell denotes the average log2(FPKM) value, in which rose red and blue signify elevated and attenuated abundance, respectively, as shown by the color scale. The identified bHLH TF (*Vitvi17g00046)* exhibited notable expression differences and high FPKM values, identifying participate in the anthocyanins accumulation.

TFs regulate anthocyanin synthesis by controlling structural gene levels in the pathway. A total of 147 TFs were identified through transcriptome annotation, primarily from the AP2/ERF, bHLH, MYB, WRKY, GATA, NAC, C2H2, and bZIP families (Figure 4). Notably, more TFs exhibited low expression levels at the early stage than the late stage, implying a complex regulation of secondary metabolites early in development, potentially involving negative regulators of anthocyanin accumulation. Among the six identified bHLH TFs, three (*Vitvi17g00046*, *Vitvi01g01745*, and *Vitvi05g01103*) were continuously upregulated, indicating their potential role as positive regulators of anthocyanin accumulation.

To ensure the reliability and consistency of RNA-Seq, 12 DEGs linked to anthocyanin accumulation were picked for qRT-PCR (Table S3). While the fold changes observed in qRT-PCR and RNA-Seq did not match precisely, their trends were consistent (Figure S3). These findings indicate that the RNA-Seq results are reliable and reproducible.

### 2.6. Expression of VvbHLH137 Correlates with Anthocyanin Accumulation in Grape Peels

**Figure 5:**
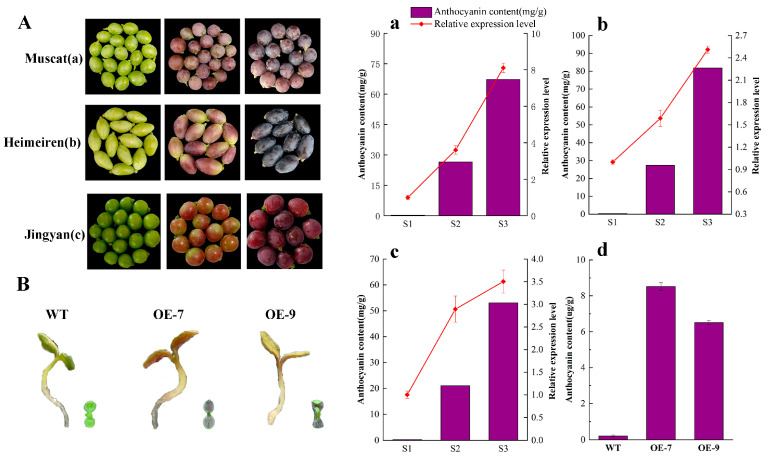
*VvbHLH137* expression and genetic function. (**A**) Variations in fruit appearance, *VvbHLH137* expression, and corresponding anthocyanin content in grape peels during developmental stages (S1 to S3) for three grape varieties. Data are means ± SDs of 3 replicates. (**B**) Over-expression of *VvbHLH137* in Arabidopsis increased anthocyanin content compared to wild-type (WT) plants. (**a**) The change of anthocyanin content and *VvbHLH137* expression in Muscat; (**b**) The change of anthocyanin content and *VvbHLH137* expression in Heimeiren; (**c**) The change of anthocyanin content and *VvbHLH137* expression in Jingyan. (**d**) The comparison of anthocyanin content in transgenic lines and WT.

The gene *Vitvi17g00046*, annotated as *VvbHLH137*, exhibited notable expression differences and high FPKM values, identifying it as a primary candidate bHLH transcription factor. *VvbHLH137*, from the berry of the ‘Moldova’ grape variety, encodes a 349 amino-acid protein of 38.75 kD with an isoelectric point of 6.41, as expected. The qRT-PCR analysis for validating *VvbHLH137* expression across the different grape varieties (Muscat, Heimeiren, Jingyan) showed that the *VvbHLH137* levels in the berries of three grape varieties gradually increased alongside anthocyanin accumulation (Figure 5A), suggesting that *VvbHLH137* is critical in anthocyanin accumulation in grape peels.

### 2.7. Over-Expression of VvbHLH137 Increases Anthocyanin Accumulation in Arabidopsis thaliana

To validate the regulatory role of *VvbHLH137* in anthocyanin accumulation, the pCAMBIA2300-*VvbHLH137* plasmid was transformed into Arabidopsis using the floral dip method, resulting in 15 transgenic lines. A PCR examination of the DNA extracted from the leaves of these lines proved the existence of the transgene. In 10-day-old stem tips of transgenic Arabidopsis, compared to WT plants, the over-expression of *VvbHLH137* showed red stems and leaves and a significantly increased anthocyanin content (Figure 5B).

### 2.8. VvbHLH137 Directly Interacts with VvMYBs

**Figure 6:**
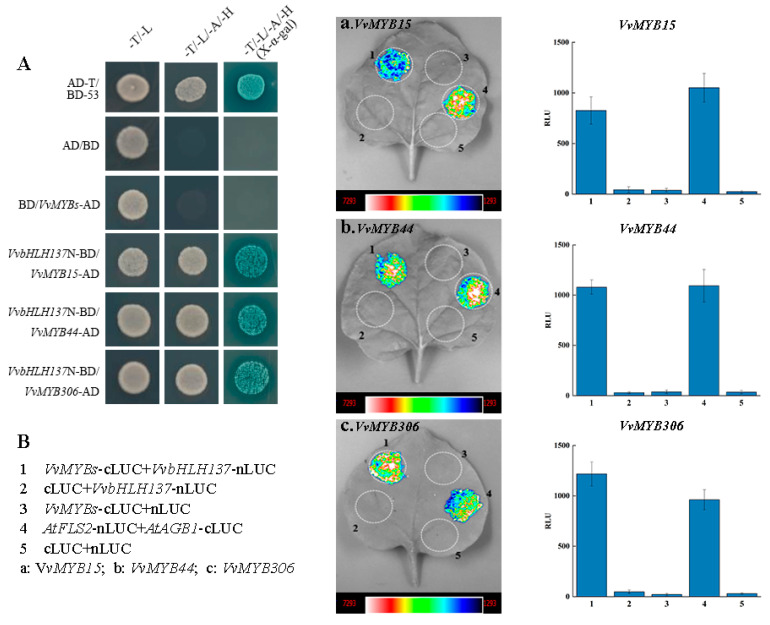
*VvbHLH137* protein interaction with *VvMYBs*. (**A**) Y2H assays showing the interaction of *VvbHLH137*N (1-760bp) with VvMYBs. Co-transformation of AD and BD plasmids served as a negative control. (**B**) LCAs confirming the interactions of *VvbHLH137* with *VvMYBs*. Agrobacterial strains *GV3101* carrying *VvMYBs*-cLUC and *VvbHLH137*-nLUC were co-infiltrated in tobacco leaves. After 3 days of incubation in the dark, bioluminescence signals were detected. Data are means ± SDs of 3 replicates. (**a**,**b**,**c**) Bioluminescence signals of interactions of *VvbHLH137* with *VvMYB15, VvMYB44, VvMYB306*.

The MYB-bHLH module complex is a key in anthocyanin synthesis. Transcriptome data identified several candidate MYB genes potentially involved in anthocyanin accumulation (Figure 4). Yeast two-hybrid (Y2H) assays were employed to explore if these *VvMYBs* communicate with *VvbHLH137*. Candidate MYB genes were introduced into pGADT7, while *VvbHLH137*N without the self-activation fragment was introduced into pGBKT7. Transformed yeast cells were cultivated at 30 °C on SD-T/-L and SD-T/-L/-H/-A media. The yeast co-transformed with *VvMYBs*-AD and *VvbHLH137*N-BD (*VvbHLH137* without the self-activation fragment) grew successfully on the SD-T/-L/-H/-A medium (Figure 6A), indicating that *VvbHLH137* communicates with *VvMYB15*, *VvMYB44*, and *VvMYB306* in yeast. To further validate these interactions, luciferase complementation assays (LCAs) were conducted in tobacco leaves. Strong bioluminescence signals were observed in combinations of *VvbHLH137*-cLUC + *VvMYB15*-nLUC, *VvbHLH137*-cLUC + *VvMYB44*-nLUC, and *VvbHLH137*-cLUC + *VvMYB306*-nLUC relative to the controls (Figure 6B). These findings unveiled the direct interaction of *VvbHLH137* with *VvMYB15*, *VvMYB44*, and *VvMYB306*.

### 2.9. Detection and Verification of the Direct Downstream Genes of VvbHLH137

**Figure 7:**
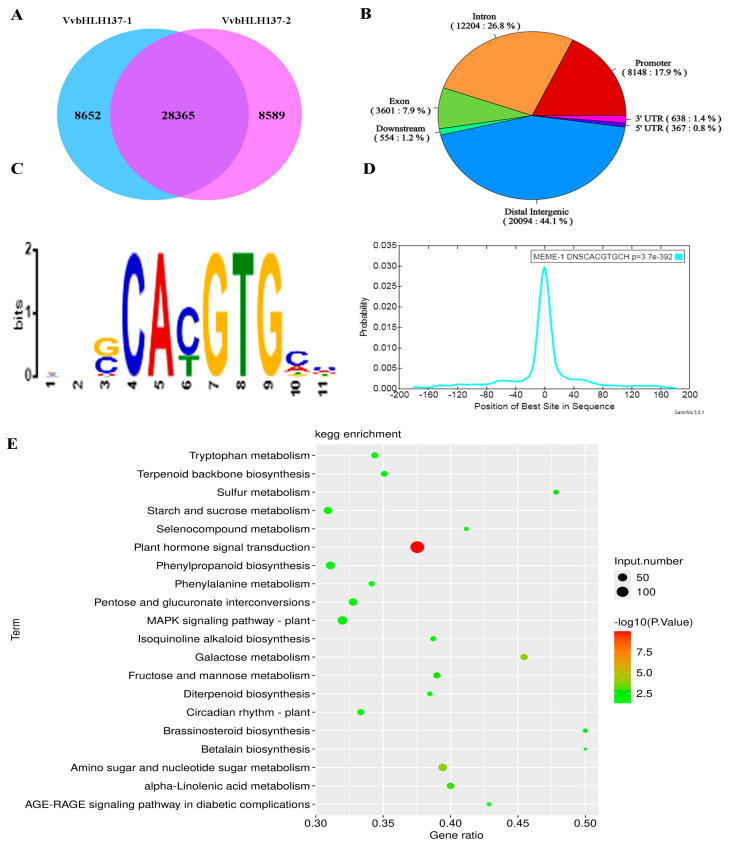
Identification and verification of genes targeted by *VvbHLH137* at the genome level. (**A**) Number of *VvbHLH137*-binding peaks, identified via DAP-seq across two technical replicates. (**B**) Genomic distribution of *VvbHLH137*-binding peaks identified with *p* < 0.05. Promoter regions were deemed as 2 kb within the upstream of the ATG start codon. (**C**) The most enriched binding motif (Motif 1) of *VvbHLH137*. (**D**) Distribution of the motif-binding positions. (**E**) Bubble plots of top 20 KEGG enrichment pathways of promoter-related genes.

To study *VvbHLH137*-mediated transcriptional regulation, DAP-seq was employed to explore *VvbHLH137* binding sites at the genome level. Among the 45,606 binding peaks identified, 28,365 were consistently present in two technical repeats (Figure 7A). Nearly half of these peaks (20,094, 44.1%) were located in the intergenic regions, while 17.9% (8148) were positioned in the promoter regions (Figure 7B). These promoter-targeted genes were associated with biological processes such as ‘Plant hormone signal transduction,’ ‘MAPK signaling pathway–plant,’ ‘Phenylpropanoid biosynthesis,’ and ‘Starch and sucrose metabolism’ (Figure 7E). The de novo motif prediction was that the predominant motif (Motif 1) contained an 11 bp palindromic conserved sequence (5′-DNSCACGTGCH-3′) (Figure 7C). The distribution analysis indicated Motif 1 was mainly located at the apex of *VvbHLH137*-binding peaks (Figure 7D), suggesting its reliability as a *VvbHLH137*-binding site in grapes.

**Figure 8:**
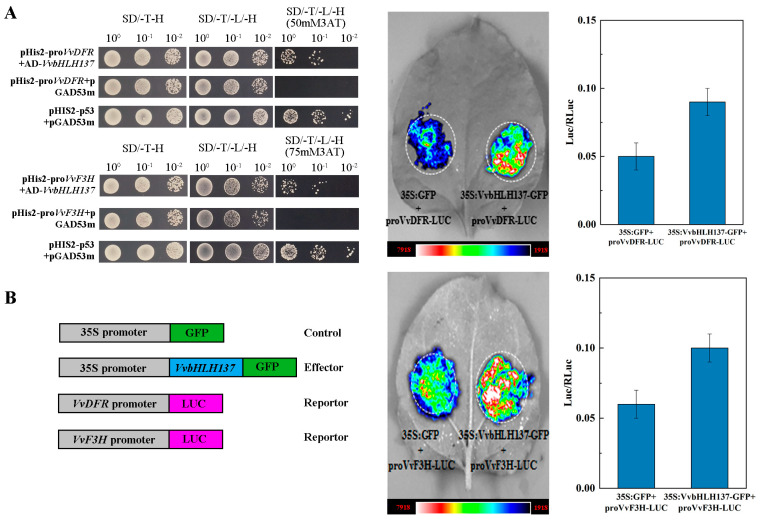
Binding of *VvbHLH137* to the promoter of *VvF3H* and *VvDFR*. (**A**) Y1H assays confirming the association of *VvbHLH137* with the promoters of *VvF3H* and *VvDFR*. (**B**) Transient LUC expression assays in tobacco leaves uncovering that LUC signals with the control of *VvF3H* and *VvDFR* promoters were enhanced by *VvbHLH137*. Relative luciferase activity was normalized to the internal control RLUC. Data are depicted as the average ± SD of three measurements.

Two anthocyanin-synthesis-related genes, *VvF3H* (*Vitvi04g01454*, encoding flavanone 3-hydroxylase) and *VvDFR* (*Vitvi18g00988*, encoding flavanone 4-reductase), were selected based on the DAP-seq data for further analysis. Y1H tests were performed to validate whether *VvbHLH137* directly binds to these genes’ promoters. Yeast cells co-transformed with *VvbHLH137*-AD+*proVvDFR*-pHis2 or *VvbHLH137*-AD+*proVvF3H*-pHis2 were cultured successfully in SD-Trp/-Leu/-His medium with 50 mM 3-AT, while those co-transformed with *VvbHLH137*-AD+*proVvF3H*-pHis2 were cultured successfully in SD (-T/-L/-H) medium supplemented with 75 mM 3-AT (Figure 8A), confirming the association of *VvbHLH137* with the promoters of *VvF3H* and *VvDFR*. Additionally, dual-luciferase reporter assays in tobacco leaves were conducted to verify *VvbHLH137*‘s regulatory function. Reporter constructs *proVvF3H*-LUC and *proVvDFR*-LUC were co-transformed with the effector 35S::*VvbHLH137*-GFP, using 35S::GFP as a control (Figure 8B). Stronger bioluminescence signals were detected in tobacco leaves co-expressing 35S::*VvbHLH137*-GFP+*proVvF3H*-LUC or 35S::*VvbHLH137*-GFP+*proVvDFR*-LUC compared to the controls, indicating that *VvbHLH137* directly augments the anthocyanin-synthesis-related genes *VvF3H* and *VvDFR*.

## 3. Discussion

As is widely known, anthocyanin content and composition vary among grape cultivars, accounting for differences in peel color [1,34,35]. For instance, white grapes lack anthocyanin accumulation. In ‘Ruby Okuyama,’ cyanidin-type anthocyanins dominate, with only minimal amounts of peonidin-type anthocyanins. Conversely, ‘Flame Muscat’ and ‘Benitaka’ are rich in peonidin-type anthocyanins, which constitute over 56% and 90% of their total anthocyanin content, respectively [36]. The predominant anthocyanins in four cultivars (cabernet sauvignon, merlot, vranec, and pinot noir) are of the malvidin type, with relative contents ranging from 35.8% in cabernet sauvignon to 67.1% in pinot noir [37]. This study further unveiled the unique anthocyanin profile of the ‘Moldova’ grape, confirming its richness in malvidin-type anthocyanins, consistent with prior findings. Notably, malvidin-type anthocyanins accounted for over 78% of the total, with malvidin-3,5-O-diglucoside and malvidin-3-O-glucoside being the most prevalent (Table S1).

TFs are crucial in regulating anthocyanin-related gene expression in plants [38,39,40]. This study unveiled 147 TFs, primarily from the AP2/ERF, bHLH, MYB, WRKY, GATA, NAC, C2H2, and bZIP families, which exhibited significant differential expression during grape berry development. This finding suggests that TFs act as key regulators of anthocyanin synthesis. Among them, the expression of eight MYB genes, including *VvMYBA1*, *VvMYB15*, *VvMYB44,* and *VvMYB306*, consistently increased from S1 to S3 (Figure 4), aligning with previous studies [36,41]. Some MYB genes negatively regulate anthocyanin synthesis, such as *TgMYB4 (Tulipa Gesneriana)* [42], *FaMYB1(Fragaria ananassa)* [43], *FtMYB18(Fagopyrum tataricum)* [44], and *PpMYB18* [45]. In apples, *MdMYB16* and its homolog *MdMYB111* inhibit anthocyanin accumulation [46,47], while *MdMYB28* over-expression suppresses anthocyanin synthesis in tobacco flower petals [48]. This study unveiled the downregulation of eight MYB genes, including *Vitvi14g00974 (VvMYB6)*, *Vitvi01g00867 (VvMYB1R1)*, and *Vitvi09g00300 (VvMYBS3)*, from S1 to S3, indicating their roles in suppressing anthocyanin accumulation.

Recent research has emphasized the role of ERF proteins in modulating anthocyanin synthesis. For example, *PpERF96* and *PbERF22* in pear (*Pyrus*) and *MdERF1*, *MdERF3*, and *MdERF38* in apple augment anthocyanin synthesis [49,50]. In mulberry *(Morus abla)*, *MaERF5* enhanced anthocyanin accumulation by communicating with Ma*MYBA* and *MaF3H* genes [51]. However, *PpERF9* was shown to subdue *PpRAP2.4* and *PpMYB114* via histone deacetylation, thereby inhibiting anthocyanin synthesis. These results unveiled that ERF TFs are critical in regulating anthocyanin synthesis. In our study, 19 DEGs encoding ERF TFs were identified, with *VvERF4* and *VvERF27* exhibiting higher expression levels (Figure 4), hinting at potential yet undefined roles in anthocyanin accumulation. Additionally, WRKY TFs are also critical for anthocyanin synthesis [52,53,54]. For example, in pears, the *PyWRKY26-PybHLH3* complex directly targets the *PyMYB114* promoter, promoting anthocyanin accumulation and leading to red skin [55]. In grapes, *VvWRKY40* interacts with *VvMYB15* and binds to the promoter of *VvF3′5′H* and *VvUFGT*, enhancing their expression and inducing anthocyanin accumulation [41]. Furthermore, *VvWRKY5* improves anthocyanin accumulation via *VvMYBA1* in response to wounding [56], and *MdWRKY40* is essential for anthocyanin accumulation in wounded apple tissues [57]. This study identified seven WRKY TFs, among which, *Vitvi05g00145* (*VvWRKY48*) showed high expression levels, warranting further investigation into its role in anthocyanin synthesis.

This study unveiled a bHLH TF, *VvbHLH137,* and observed that its levels increased during peel coloration across different grape cultivars (Figure 5). Additionally, *VvbHLH137*‘s upregulation in Arabidopsis plants augmented anthocyanin levels, demonstrating that *VvbHLH137* is essential in berry coloration. bHLH TFs bind to specific MYB proteins to modulate downstream genes responsible for anthocyanin synthesis [10,23]. Based on our findings and previous research, we propose that *VvbHLH137* interacts with key MYB regulators, including *VvMYBA1* [58], *VvMYB15*, *VvMYB44*, and *VvMYB306*, to promote anthocyanin accumulation (Figure 6).

Evidence also suggests that bHLH proteins directly interact with the structural gene promoters to modulate anthocyanin synthesis. For example, *CsbHLH89* promotes anthocyanin synthesis in tea via interacting with G-box elements in the promoters of *CsCHS*, *CsFLS*, and *CsDFR* [24]. Similarly, *MdbHLH3* interacts with the *MdDFR* promoter to facilitate fruit reddening in apples [21]. This study demonstrated that *VvbHLH137* interacts with and activates *VvDFR* and *VvF3H* promoters, as confirmed by Y1H and dual-luciferase assays (Figure 8). These outcomes implied that *VvbHLH137* is crucial in grape peel anthocyanin synthesis by directly associated with promoters to regulate the expression of *VvDFR* and *VvF3H.* Overall, our study unveils key insights into *VvbHLH137* regulation in grape berry color formation and lays the foundation for understanding color pattern development in grape cultivars.

## 4. Materials and Methods

### 4.1. Plants

The Moldova grape (*Vitis labrusca* × *Vitis vinifera*) cultivar was planted in a ‘single-armed dragon trunk + v-shaped leaf curtain’ system under standard cultivated conditions in the greenhouse of Shijiazhuang Institute of Pomology, Hebei Academy of Agriculture and Forestry Sciences (Shijiazhuang, Hebei Province, China). The daily management schedule was implemented according to standardized grape production. The berry color change for Moldova grapes occurred approximately 65 days after anthesis (DAA). Therefore, berries were collected at green (51 DAA), veraison (65 DAA), and maturity (79 DAA) stages. The harvested berries were washed with 75% alcohol, and the peels were separated using a scalpel. Equal amounts of peel tissue were gathered in three sets as biological replicates. The samples were rapidly chilled in liquid nitrogen and preserved at −80 °C for extended examination.

### 4.2. Anthocyanin Content Determination

Total anthocyanin was determined using a plant anthocyanin test kit (Comin Biotechnology Co., Ltd., Suzhou, China). In short, anthocyanin was retrieved from 0.1 g sample at 75 °C in 1 mL extraction solution, which was shaken for 25 min. The supernatant was harvested after 10 min of spinning at 12,000 rpm at room temperature for anthocyanin detection. The supernatant was dissolved into solution 1 and solution 2 and simultaneously tested for absorbance at 530 nm and 700 nm, then the total amount of anthocyanins was calculated. Individual anthocyanins were quantified using UPLC-MS/MS. Filtered extracts (2 μL) were loaded on a UPLC column (2.1 × 100 mm ACQUITY UPLC BEH C18 column with 1.7 μm particles) at 0.35 mL/min and separated under a gradient program using solvent A (0.1% formic acid in water) with solvent B (0.1% formic acid in acetonitrile) from 5% for 5 min to 50% for over 6 min to 95% for over 2 min. Mass spectrometry (MS) was executed in positive ion mode using an electrospray ionization (ESI) interface with nebulizer pressure of 35 psi, N_2_ as drying gas at 10 L/min and 400 °C, and scan range of 150–1200 *m*/*z*. Based on the standard database, the mass spectrometry data were qualitatively analyzed, while the chromatogram was integrated using the standard curve. All samples were analyzed with three replicate samples.

### 4.3. RNA Isolation and Sequencing

Samples were snap-frozen and pulverized into a refined powder in liquid nitrogen. Total RNA was prepared with the Eastep^®^ Super Total RNA Extraction Kit (Promega Co., Ltd., Shanghai, China) and used to construct sequencing libraries. After quality assurance, the library was sequenced at Novogene Co., Ltd., Beijing, China. Clean reads were produced after eliminating poor-quality reads based on GC content distribution and sequencing error rate. Subsequently, clean reads were aligned using HISAT2 software to the *V. vinifera* genome from the EnsemblPlants database (http://plants.ensembl.org/Vitis_vinifera/Info/Index, accessed on 5 September 2023) (v2.1.0) [59], providing their locations on the reference, and charted to each gene using the subread package in featureCounts software (v2.0.3) to examine gene levels by computing their FPKMs [60] (fragments per kilobase of transcript sequence per million base pairs sequenced). Additionally, DEGs were detected using DESeq2 [61] based on |log2FoldChange| > 1 and *p*-value ≤ 0.05 and underwent Gene Ontology and Kyoto Encyclopedia of Genes and Genomes (KEGG) pathway enrichment analyses using clusterProfiler software (v4.6.0) [62].

### 4.4. Quantitative Real-Time PCR (qRT-PCR)

To confirm transcription levels, the total RNA used for sequencing was subjected to qRT-PCR. Briefly, samples were converted to cDNA using HiScript III RT SuperMix (Vazyme, Nanjing, China) and analyzed with three biological and technical replicates on a Light Cycler 96 Real-Time PCR Detection System (Roche, Basel, Switzerland) using the SYBR Green PCR Master Mix (TaKaRa, Osaka, Japan). The reaction program included activation at 95 °C for 10 min, followed by 45 cycles of 95 °C for 10 s, 58 °C for 10 s, and 68 °C for 10 s. Primers were created using Primer Premier 5.0 and are listed in Table S1. Actin (LOC100232866) was employed as a control (Table S3) to calculate relative expression using the 2^−△△Ct^ algorithm [63].

### 4.5. Stable Transformation of Arabidopsis

The pCAMBIA2300-*VvbHLH137* construct was introduced into Agrobacterium tumefaciens *GV3101* employing the freeze–thaw method. Positive clones were cultured in LB medium (50 μg/mL kanamycin + 30 μg/mL rifampicin) to OD600 = 0.8 and centrifugated. The precipitation was resuspended using MS medium (100 μmol/L acetosyringone + MS + 20 g/L sucrose) and stewed at 28 °C in the dark for 2 h before infiltration. Stable transformation of Arabidopsis *Col-0* was achieved using the floral dip method. Transformants were cultured on MS basal medium with 50 μg/mL kanamycin and confirmed using PCR. T3 transgenic lines were subjected to phenotyping. Wild-type Arabidopsis *Col-0* and *VvbHLH137* transgenic T3 seeds were embedded in MS medium with 2X sucrose (40 g/L). Color observation and anthocyanin detection were performed 10 days later.

### 4.6. Yeast Two-Hybrid (Y2H) Assays

bHLH TFs often form complexes with MYB TFs to activate anthocyanin synthesis in plants. Based on transcriptome analysis, potential MYB TFs were screened out. Given current study and the expression level analysis in the transcriptome, we chose *VvMYB15*, *VvMYB44*, and *VvMYB306* as our candidate genes. For Y2H assays, the coding sequences (CDSs) of *VvbHLH137* and *VvMYBs* were cloned into BamHI and EcoRI-predigested pGBKT7 with DNA-binding domain BD and pGADT7 with transcriptional activation domain AD to generate *VvMYBs*-AD and *VvbHLH137*-BD, respectively. The *VvMYBs*-AD was transformed independently or jointly with *VvbHLH137*-BD into the yeast strain Y2H Gold using the PEG/LiAc method. Autoactivation of *VvbHLH137* was assessed. Transformants were screened on a double-dropout medium (SD-Leu/-Trp) using yeast colony PCR, and positive samples were cultivated on the quadruple-dropout medium (SD-Leu/-Trp/-His/-Ade) to affirm associations by assessing α-galactosidase activity using X-α-Gal (200 ng/mL).

### 4.7. Luciferase Complementation Assays (LCAs)

To assess the interactions between *VvbHLH137* and MYB TFs, the CDSs of *VvbHLH137* and *VvMYBs* were introduced into pCAMBIA1300-nLUC and pCAMBIA1300-cLUC vectors, respectively. The resulting recombinants (*VvbHLH137*-nLUC and *VvMYBs*-cLUC) were transferred into Agrobacterium tumefaciens strain *GV3101* using the freeze–thaw protocol. Tobacco leaves were infiltrated with an Agrobacterium suspension (OD600 = 0.6) containing the recombinant vectors. Two days post-infiltration, luciferase activity was analyzed using a Tanon-5200 plant visualization system (Tanon, Shanghai, China) after sprayed 100uM concentration of D-luciferin. The interaction intensity between samples was quantified using a microplate reader. Primers are provided in Table S1.

### 4.8. DNA Affinity Purification Sequencing (DAP-Seq)

DAP-seq was conducted as depicted [64]. Genomic DNA was prepared from Moldova plant leaves and converted into a library using the MICH TLX DNA-seq kit (PerkinElmer, Austin, TX, USA). The *VvbHLH137* proteins were synthesized using the TNT SP6 Coupled Wheat Germ Extract System (Promega, Madison, WI, USA) and pulled down with HaloTag beads (Promega, USA). The mixture was reacted with the library, while HaloTag beads alone were used as a negative control. DNAs bound to *VvbHLH137* were eluted and sequenced on an Illumina NavoSeq6000 (San Diego, CA, USA). Sequencing reads were blasted against the grape reference genome (*Vitis vinifera*) (http://plants.ensembl.org/Vitis_vinifera/Info/Index, accessed on 27 June 2024) using BWA-MEM [65]. DAP-seq peaks were extracted with MACS2 [66] based on fold enrichment > 2 and *p*-value < 0.05. Conserved motifs in the identified peaks were assessed using MEME-CHIP software (version 5.0.5) [67].

### 4.9. Yeast One-Hybrid (Y1H) Assays

The DNA were prepared with the Fastpure Plant DNA Isolation Kit (Nanjing Vazyme Biotech Co., Ltd., Nanjing, China). The promoters of *VvF3H* and *VvDFR* were cloned and introduced into XhoI- and EcoRI-predigested pHis2, while the *VvbHLH137* CDSs were introduced into pGADT7. To decide the optimal 3-amino-1, 2, 4-triazole (3AT) concentration for suppressing background histidine leakiness, two recombinant pHis2 vectors were transformed into the yeast strain Y187 and grown on SD-Trp/-His/-Leu medium with varying 3AT levels. For the Y1H assay, the pHis2 recombinant vectors and pGADT7 recombinant vectors were co-transformed into yeast Y187. Negative controls included co-transformation of empty pGADT7 with the pHis2 recombinant vectors. Transformed Y187 cells were plated on SD-Trp/-His/-Leu medium with the optimal 3AT concentration, and colonies were monitored to assess the interaction of *VvbHLH137* with *VvF3H* and *VvDFR* promoters.

### 4.10. Transient Luciferase Expression Assays in Tobacco

The transient luciferase expression assay was executed as below: the promoter regions of *VvF3H* and *VvDFR* were cloned into the pGreenII0800 vector to create *ProVvF3H*:LUC and *ProVvDFR*:LUC reporter vectors, respectively. These vectors were co-infiltrated into tobacco leaves with the effector constructs 35S::*VvbHLH137*-GFP or 35S::GFP. Renilla luciferase (RLUC) was included to normalize the data. The relative luciferase activity was calculated as the LUC/RLUC ratio.

## 5. Conclusions

This study revealed that malvidins, particularly malvidin-3,5-O-diglucoside and malvidin-3-O-glucoside, are the predominant anthocyanins in Moldova grapes. Through transcriptome sequencing, numerous DEGs linked to anthocyanin accumulation in grape peels were unveiled, providing a foundational understanding of the gene regulation mechanisms underlying the color development of Moldova grapes. Additionally, we demonstrated that *VvbHLH137*, a bHLH transcription factor, interacts with *VvMYBA1*, *VvMYB15*, *VvMYB44*, and *VvMYB306* to regulate anthocyanin synthesis and accumulation. This is achieved through the direct activation of the promoters of *VvDFR* and *VvF3H*. These findings advance our knowledge of anthocyanin synthesis regulation in grapes and support molecular breeding efforts to develop grape cultivars with desirable coloration.

## Data Availability

All data supporting the findings of this study are available within the paper and the Supplementary Data section published online.

## Supplementary Materials

The following supporting information can be downloaded at: https://www.mdpi.com/article/10.3390/plants14060871/s1. Figure S1. GO enrichment analysis; Figure S2. KEGG enrichment analysis; Figure S3. qRT-PCR validation of the 12 genes related to anthocyanin biosynthesis in the transcriptome. The relative expression levels of 12 DEGs were analyzed by qRT-PCR and Actin was used as reference gene. The specific primers of tested genes were listed in Table S3. Data are presented as means ± standard errors (±SE) (n = 3). Figure S4. auto-activation of VvbHLH137; Table S1. Statistics of the detected substances in the Moldova grape peel of three states; Table S2. Basic information on transcriptome sequencing; Table S3. Primer summary information.
