# Supplementary material for: Transcription Factor VvbHLH137 Positively Regulates Anthocyanin Accumulation in Grape (Vitis vinifera)
Source: Plants (Basel). 2025 Mar 11;14(6):871. doi: 10.3390/plants14060871 (PMC11946382; doi:10.3390/plants14060871)
Supplement: Supplementary file 1 [file plants-14-00871-s001.zip › Figure S3. qRT-PCR validation of the 12 genes related to anthocyanin biosynthesis in the transcriptome.pdf]

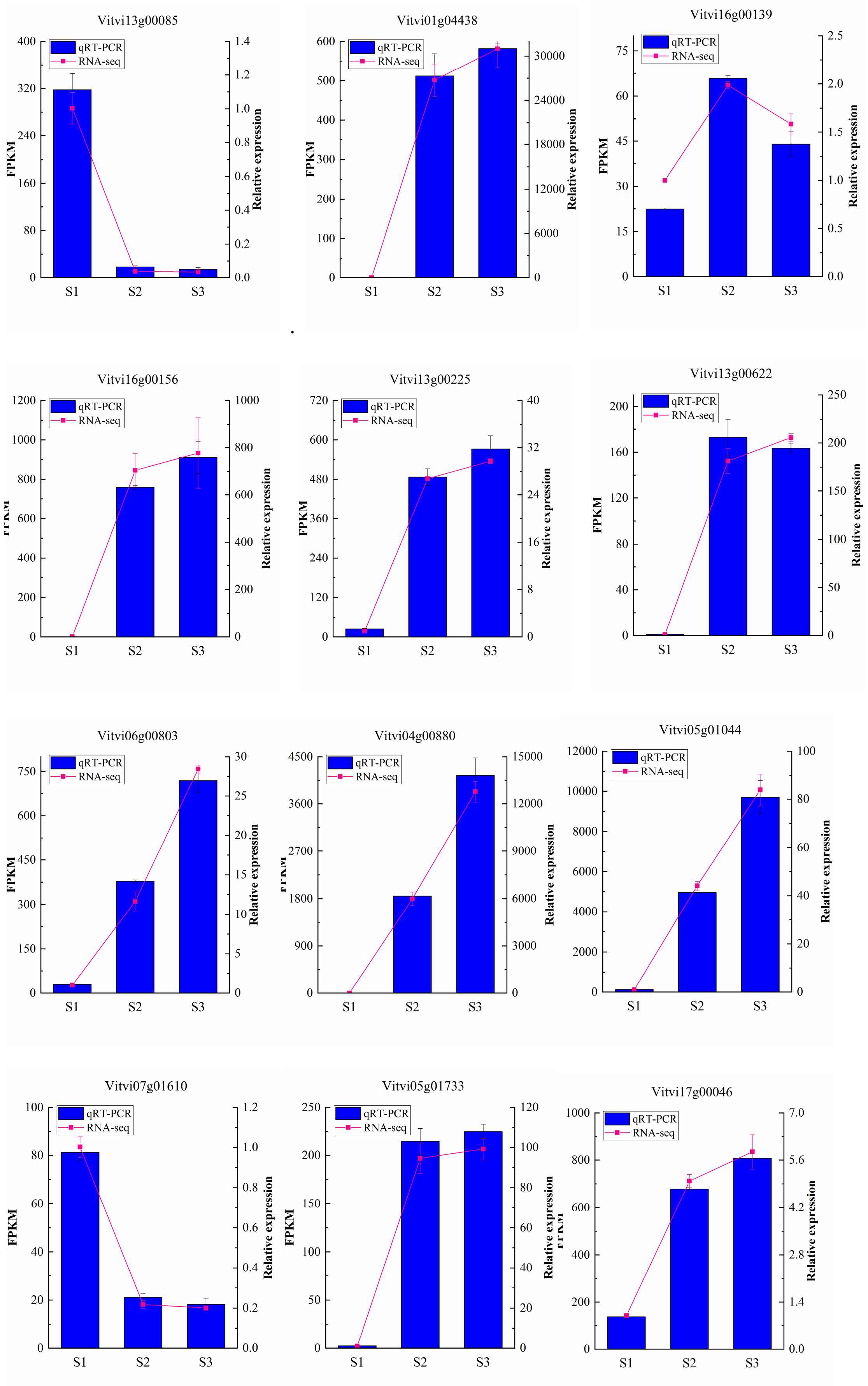
Vitvi13g00085
Vitvi01g04438
Vitvi16g00139
Vitvi16g00156
Vitvi13g00225
Vitvi13g00622
Vitvi06g00803
Vitvi04g00880
Vitvi05g01044
Vitvi07g01610
Vitvi05g01733
Vitvi17g00046
qRT-PCR
RNA-seq
FPKM
Relative expression
S1
S2
S3

Figure S3. qRT-PCR validation of the 12 genes related to anthocyanin biosynthesis in the transcriptome. The relative expression levels of 12 DEGs were analyzed by qRT-PCR and Actin was used as reference gene.The specific primers of tested genes were listed in Table S3. Data are presented as means ± standard errors (± SE) (n=3).
