# Supplementary material for: Transcription Factor VvbHLH137 Positively Regulates Anthocyanin Accumulation in Grape (Vitis vinifera)
Source: Plants (Basel). 2025 Mar 11;14(6):871. doi: 10.3390/plants14060871 (PMC11946382; doi:10.3390/plants14060871)
Supplement: Supplementary file 1 [file plants-14-00871-s001.zip › Table S1. Statistics of the detected substances in the Moldova grape peel of three states.pdf]

Table S1. Statistics of the detected substances in the Moldova grape peel of three states

Compounds	Class	Molecular Weight	Formula	S1-1	S1-2	S1-3	S2-1	S2-2	S2-3	S3-1	S3-2	S3-3
Cyanidin-3,5-O-diglucoside	Cyanidin	611.16	C27H31O16+	0.06	0.06	0.06	5.54	7.80	8.14	47.55	45.77	40.06
Cya-3-O-sophoroside-5-O-glucoside	Cyanidin	773.21	C33H41O21+	N/A	N/A	N/A	N/A	N/A	N/A	0.00	0.00	0.00
Cyanidin-3-O-arabinosidase-glucoside	Cyanidin	581.15	C26H29O15+	N/A	N/A	N/A	N/A	N/A	N/A	0.07	0.06	0.06
Cyanidin-3-O-(acetyl)galactoside	Cyanidin	491.12	C23H23O12+	N/A	N/A	N/A	N/A	N/A	N/A	0.09	0.09	0.07
Cyanidin-3-(ferulyl)glucoside	Cyanidin	625.16	C31H29O14+	N/A	N/A	N/A	N/A	N/A	N/A	0.02	0.02	0.01
Cyanidin-3-O-xyloside	Cyanidin	419.10	C20H19O10+	N/A	N/A	N/A	0.02	0.02	0.02	0.24	0.26	0.20
Cyanidin-3-O-(6"-O-caffeoyl)rhamnoside	Cyanidin	595.15	C30H27O13+	N/A	N/A	N/A	0.44	0.63	0.58	0.28	0.30	0.22
Cyanidin-3-O-glucoside	Cyanidin	449.11	C21H21O11+	N/A	N/A	N/A	8.12	10.71	10.41	45.20	45.82	39.61
Delphinidin-3-(6"-O-coumaroyl)galactoside)	Delphinidin	611.14	C30H27O14+	N/A	N/A	N/A	N/A	N/A	N/A	0.11	0.12	0.08
Delphinidin-3-O-(6"-O-xylosyl)glucoside	Delphinidin	597.15	C26H29O16+	0.03	0.03	0.03	N/A	N/A	N/A	N/A	N/A	N/A
Delphinidin-3-O-rhamnoside	Delphinidin	449.11	C21H21O11+	N/A	N/A	N/A	N/A	N/A	N/A	0.00	0.00	0.00
Delphinidin-3-O-(6"-O-caffeoyl)glucoside	Delphinidin	627.13	C30H27O15+	0.04	0.03	0.02	0.04	0.05	0.04	N/A	N/A	N/A
Delphinidin-3-O-glucoside	Delphinidin	465.10	C21H21O12+	N/A	N/A	N/A	28.51	41.69	41.71	471.99	466.78	417.63
Delphinidin-3,5-O-diglucoside	Delphinidin	627.16	C27H31O17+	N/A	N/A	N/A	49.94	69.72	73.92	519.54	555.68	473.12
Delphinidin-coumaroyl-Sophorotriose	Delphinidin	935.25	C42H47O24+	N/A	N/A	N/A	0.01	0.02	0.02	0.01	0.01	0.01
Delphinidin-3-O-(6-O-malonyl-beta-D-glucoside)	Delphinidin	551.10	C24H23O15+	0.07	0.05	0.08	0.06	0.07	0.06	0.05	0.04	0.05
Delphinidin-3-O-arabinoside	Delphinidin	435.09	C20H19O11+	0.07	0.06	0.06	0.05	0.06	0.06	0.02	0.02	0.02
Delphinidin-3-O-(6-O-malonyl)-glucoside-3'-glucoside	Delphinidin	713.16	C30H33O20+	0.04	0.03	0.04	0.03	0.06	0.04	0.05	0.06	0.07
Malvidin-3-O-glucoside	Malvidin	493.13	C23H25O12+	N/A	N/A	N/A	163.77	214.72	225.08	2348.96	2346.53	2083.34
Malvidin-3-O-(6-O-p-coumaroyl)-glucoside	Malvidin	639.17	C32H31O14+	N/A	N/A	N/A	6.52	9.54	9.60	79.30	80.58	78.31
Malvidin-3,5-O-diglucoside	Malvidin	655.19	C29H35O17+	N/A	N/A	N/A	1207.69	1531.73	1501.39	8030.44	7784.16	7366.37
Malvidin-3-O-glucoside-5-O-galactoside	Malvidin	655.19	C29H35O17+	N/A	N/A	N/A	N/A	N/A	N/A	2.71	2.34	1.92
Malvidin-3-O-(6"-O-feruloyl)glucoside	Malvidin	669.18	C33H33O15+	N/A	N/A	N/A	N/A	N/A	N/A	1.01	1.03	0.86

Malvidin-3-O-(6"-acetylglucoside)-5-glucoside	Malvidin	697.20	C31H37O18+	0.06	0.06	0.04	40.36	53.67	55.33	561.09	546.62	493.73
Malvidin-3-O-(6"-O-caffeoyl)glucoside	Malvidin	655.17	C32H31O15+	N/A	N/A	N/A	0.20	0.28	0.24	2.71	2.96	1.82
Malvidin-3-O-arabinoside	Malvidin	463.12	C22H23O11+	N/A	N/A	N/A	N/A	N/A	N/A	0.02	0.02	0.03
Malvidin-3-O-(6"-O-malonyl)galactoside	Malvidin	579.13	C26H27O15+	N/A	N/A	N/A	N/A	N/A	N/A	0.00	0.00	0.00
Malvidin-3-O-(6"-O-sinapoyl)xyloside	Malvidin	669.18	C32H33O15+	N/A	N/A	N/A	0.02	0.03	0.03	1.01	1.02	0.82
Malvidin-3-O-(6"-O-coumaroyl)rhamnoside	Malvidin	623.18	C32H31O13+	N/A	N/A	N/A	N/A	N/A	N/A	0.08	0.07	0.05
Pelargonidin-3-O-rhamnoside-5-O-glucoside	Pelargonidin	579.17	C27H31O14+	0.18	0.13	0.14	0.11	0.11	0.17	0.10	0.08	0.07
Pelargonidin-3,5-O-diglucoside	Pelargonidin	595.17	C27H31O15+	N/A	N/A	N/A	0.94	1.20	1.34	11.92	11.97	9.49
Pelargonidin-3-O-glucoside	Pelargonidin	433.11	C21H21O10+	N/A	N/A	N/A	0.08	0.10	0.11	0.93	0.96	0.79
Peonidin-3,5-diglucoside	Peonidin	625.18	C28H33O16+	N/A	N/A	N/A	N/A	N/A	N/A	0.48	0.47	0.39
Peonidin-3-O-(6-O-p-coumaroyl)-glucoside	Peonidin	609.16	C31H29O13+	0.01	0.01	0.01	1.97	2.97	2.81	3.21	3.47	2.73
Peonidin-3-O-glucoside	Peonidin	463.12	C22H23O11+	N/A	N/A	N/A	10.41	14.23	14.46	58.58	58.46	53.96
Peonidin-3-O-galactoside	Peonidin	463.12	C22H23O11+	0.06	0.06	0.05	N/A	N/A	N/A	N/A	N/A	N/A
Peonidin-3-O-(6"-O-acetyl-malonyl)glucoside	Peonidin	591.13	C27H27O15+	0.05	0.04	0.04	0.03	0.03	0.03	0.02	0.02	0.02
Peonidin-3-O-(acetyl)(malonyl)galactoside	Peonidin	591.13	C27H27O15+	0.05	0.04	0.04	0.06	0.07	0.07	0.03	0.03	0.03
Peonidin-3,5-O-diglucoside	Peonidin	625.18	C28H33O16+	N/A	N/A	N/A	139.57	189.68	198.05	935.24	930.91	755.45
Peonidin-3-O-araboside-glucoside	Peonidin	595.17	C27H31O15+	N/A	N/A	N/A	0.10	0.12	0.13	1.15	1.16	0.95
Peonidin	Peonidin	301.07	C16H13O6+	N/A	N/A	N/A	0.05	0.06	0.05	0.45	0.49	0.38
Petunidin-3-O-(6-O-malonyl-beta-D-glucoside)	Petunidin	565.12	C25H25O15+	0.00	0.00	0.00	0.01	0.01	0.01	0.00	0.00	0.00
Petunidin-3-O-arabinoside	Petunidin	449.11	C21H21O11+	N/A	N/A	N/A	N/A	N/A	N/A	0.02	0.02	0.01
Petunidin-3-O-glucoside	Petunidin	479.12	C22H23O12+	N/A	N/A	N/A	39.69	54.56	57.11	766.34	776.81	628.63
Petunidin-3-O-(caffeoyl)diglucoside	Petunidin	803.20	C37H39O20+	N/A	N/A	N/A	N/A	N/A	N/A	0.06	0.05	0.04
Petunidin-3-O-(6-O-p-coumaroyl)-glucoside	Petunidin	625.16	C31H29O14+	N/A	N/A	N/A	0.87	1.19	1.24	4.13	4.49	3.29
Procyanidin B3	Procyanidin	578.14	C30H26O12	39.61	34.20	38.27	38.06	47.11	45.36	31.18	30.40	25.75
Procyanidin C1	Procyanidin	866.21	C45H38O18	17.67	10.71	12.25	9.44	10.50	10.73	3.25	2.54	2.40
Procyanidin B2	Procyanidin	578.14	C30H26O12	68.93	56.04	57.82	37.29	50.79	54.35	21.36	19.32	18.94

Procyanidin B4	Procyanidin	578.14	$C_{30}H_{26}O_{12}$	9.75	8.01	8.85	6.29	8.75	8.82	3.70	4.52	3.69
Procyanidin B1	Procyanidin	578.14	$C_{30}H_{26}O_{12}$	506.37	408.32	413.67	330.63	398.99	419.60	260.48	245.24	208.24
Quercetin-3-O-glucoside	flavonoid	464.10	$C_{21}H_{20}O_{12}$	6.41	4.97	4.94	17.21	20.70	30.10	17.58	14.06	12.51
Naringenin	flavonoid	272.07	$C_{15}H_{12}O_5$	0.04	0.03	0.04	0.18	0.30	0.24	0.16	0.18	0.13
