# Supplementary material for: Transcription Factor VvbHLH137 Positively Regulates Anthocyanin Accumulation in Grape (Vitis vinifera)
Source: Plants (Basel). 2025 Mar 11;14(6):871. doi: 10.3390/plants14060871 (PMC11946382; doi:10.3390/plants14060871)
Supplement: Supplementary file 1 [file plants-14-00871-s001.zip › Table S2. Basic information on transcriptome sequencing.pdf]

Table S2. Basic information on transcriptome sequencing

Sample	Raw Reads	Clean Reads	Clean Base(G)	Reads mapped	Unique mapped	Q20(%)	Q30(%)
S1-1	49610184	48215370	7.23	45330915(94.02%)	43703025(90.64%)	97.77	93.37
S1-2	40032096	38917012	5.84	36612631(94.08%)	35361909(90.86%)	97.79	93.41
S1-3	40820880	39873566	5.98	37411970(93.83%)	36208604(90.81%)	97.7	93.15
S2-1	45252960	44089080	6.61	41598978(94.35%)	40023512(90.78%)	97.78	93.4
S2-2	51659122	49904040	7.49	47045167(94.27%)	45210382(90.59%)	97.86	93.58
S2-3	50484816	48917982	7.34	46219142(94.48%)	44527692(91.03%)	97.9	93.69
S3-1	51286402	49850782	7.48	46708283(93.70%)	44827597(89.92%)	97.81	93.5
S3-2	40998180	39786394	5.97	37366237(93.92%)	36141889(90.84%)	97.84	93.52
S3-3	46881992	45428876	6.81	42376349(93.28%)	40802991(89.82%)	97.5	92.75
