# Supplementary material for: Transcription Factor VvbHLH137 Positively Regulates Anthocyanin Accumulation in Grape (Vitis vinifera)
Source: Plants (Basel). 2025 Mar 11;14(6):871. doi: 10.3390/plants14060871 (PMC11946382; doi:10.3390/plants14060871)
Supplement: Supplementary file 1 [file plants-14-00871-s001.zip › Table S3. Primer summary information.pdf]

Table S3. Primer summary information

Primer information qRT-PCR	
Actin	Fowerd：GTGCCTGCCATGTATGTTGCC<br>Right：GGTCACGTCCAGCAAGGTCAAG
Vitvi17g00046 (*VvbHLH137*)	Fowerd：TCTTTGCAGCTTTGGACCCC<br>Right：ACTGGAGAGGTGGTCCTGTT
Vitvi13g00622 (*PAL*)	Fowerd：CACAGGTTTCGGTGCTACCT<br>Right：AGTGTGTGGCAAGATTCCGT
Vitvi16g00139 (*4CL*)	Fowerd：GGGAGACGTCGTCATGATCC<br>Right：AAGTGTAGAAGGGGTTGGCG
Vitvi06g00803(*CYP73A*)	Fowerd：GAACCACCTGAACCTCTCCG<br>Right：ATCCGAACTCCACTCCCTGA
Vitvi05g01044 (*VvCHS2*)	Fowerd：TGACACTCACTTGGACAGCC<br>Right：GTAAGTGGAAGGTCAGCCCC
Vitvi13g00225 (Vv*CHI2*)	Fowerd：GACTGTGGAGGAGTTAGCGG<br>Right：AATACTGGCGACCCGTCAAA
Vitvi16g00156 (*UFGT*)	Fowerd：AAACTCGCTCTTCTCACGCA<br>Right：GGCGGTGGGGTTATTAGGTT
Vitvi04g00880 (*GST*)	Fowerd：CTACCTCGAATGGGTGAGCG<br>Right：GTAACCAAGTGCGCCATTCC
Vitvi01g04438 (*VvAOMT*)	Fowerd：TCCAGCTCAAGTCATAGGGGC<br>Right：CCCTCATCGACAGGGACATTC
Vitvi13g00085 (*VvLOB21*)	Fowerd：ATGAGCCCCGTTCAAGTTCC<br>Right：TGGCACCAAACACCTTGTGA
Vitvi07g01610 (*VvLOB38*)	Fowerd：GCCGAGGTAACTTGTACGGA<br>Right：TAACTCATCGCTCCCAAGCC
Vitvi05g01733 (*VvMYB15*)	Fowerd：AGTTGCAGACTCCGATGGAT<br>Right：GGTGTGTGTGCCAGACATTT

Primer information of Stable transformation of Arabidopsis	
pCAMBIA2300-*VvbHLH137*	Fowerd: ggacgagctcggtacccggggatccATGGCAGCCTTTTCGTATCAACAC<br>Right：tcgcccttgctcaccatggtgtcgacTTAATTGAAAGAACACAAGTTG

Primer information of Y2H and LCA	
pGBKT7-*VvBHLH137*	Foweerd:tggccatggaggccgaattcATGGCAGCCTTTTCGTATCAACAC<br>Right: cgctgcaggtcgacggatccTTAATTGAAAGAACACAAGTTG
pGBKT7-*VvbHLH137-N*	Foweerd:tggccatggaggccgaattcATGGCAGCCTTTTCGTATCAACACCC<br>Right:cgctgcaggtcgacggatccCATCTAGGTCCATGCCAAAGTC
pGADT7-*VvMyb15*	Foweerd:gccatggaggccagtgaattcATGGTAAGAGCTCCTTGTTGT<br>Right: cagctcgagctcgatggatccTCAAAGCTCCTGTAAGCCGCCAGATC
pGADT7-*VvMyb44*	Foweerd：gccatggaggccagtgaattcATGGCGTCTTCCAAGAAAGATTTAG<br>Right：cagctcgagctcgatggatccCTACTCGATTTTGCCAATTCCGAT
pGADT7-*VvMyb306*	Foweerd：gccatggaggccagtgaattcATGGGAAGACCACCTTGCTGTGATAA<br>Right：cagctcgagctcgatggatccTCAGAAAAAATCAGGACTTTCATCTAA
pGADT7-*VvbHLH137*	Foweerd：gccatggaggccagtgaattcATGGCAGCCTTTTCGTATCAACAC<br>Right：cagctcgagctcgatggatccTTAATTGAAAGAACACAAGTTG
VvbHLH137-Nluc	Foweerd：cggtacccgggatccaATGGCAGCCTTTTCGTATCAACAC<br>Right：acgagatctggtcgacATTGAAAGAACACAAGTTGTTGCTGAT
VvMYB15-Cluc	Foweerd：cggtacccgggatccaATGGTAAGAGCTCCTTGTTGT<br>Right：agctctgcaggtcgactcaTCAAAGCTCCTGTAAGCCGCCAGATCTA
VvMYB44-Cluc	Foweerd：cggtacccgggatccaATGGCGTCTTCCAAGAAAGATTTAG<br>Right：agctctgcaggtcgactcaCTACTCGATTTTGCCAATTCCGAT
VvMYB306-Cluc	Foweerd：cggtacccgggatccaATGGGAAGACCACCTTGCTGTGATAA<br>Right：agctctgcaggtcgactcaTCAGAAAAAATCAGGACTTTCATCTAA

Primer information of Y1H and Transient luciferase expression assays in tobacco	
pHIS2-F3’Hpro	Foweerd：tacgactcactatagggcgaattcGATCAATAATGGACTAAAATGGTTGAC<br>Right：attcgcgaacgcgtgagctcGGACGTCGCCTTCTTTCTCTATCAGAC
pHIS2-DFRpro	Foweerd：tacgactcactatagggcgaattcATCCACTAAGGAGCAGAAAGCAG<br>Right：attcgcgaacgcgtgagctcACTTCTCAAAAATGATCCGGG
PCAMBIA1302-*VvBHLH137*	Foweerd：<br>ggagagaacacgggggactcttgaccATGGCAGCCTTTTCGTATCAACACCCACCTTTTCTTCTTGAC<br>Right：<br>CTCCAGTGAAAAGTTCTTCTCCTTTACTATTGAAAGAACACAAGTTGTTGCTGATGAGTCCAGATGGATTAATG
pGreenII0800-F3’Hpro	Foweerd：<br>GCGTAATACGACTCACTATAGGGCGAATTGGGTACCGATCAATAATGGACTAAAATGGTTGAC

	Right：<br>GAATGGCGCCGGGCCTTTCTTTATGTTTTTGGCGTCTTCCATGGGGAC<br>GTCGCCTTCTTTCTCTATCAGAC
pGreenII0800-<br>DFRpro	Fowerd：<br>GCGTAATACGACTCACTATAGGGCGAATTGGGTACCATCCACTAAGG<br>AGCAGAAAGCAG<br>Right：<br>GAATGGCGCCGGGCCTTTCTTTATGTTTTTGGCGTCTTCCATGGACTT<br>CTCAAAAATGATCCGGG
