# Supplementary figures and images for: Transcription Factor VvbHLH137 Positively Regulates Anthocyanin Accumulation in Grape (Vitis vinifera)
Source: Plants (Basel). 2025 Mar 11;14(6):871. doi: 10.3390/plants14060871 (PMC11946382; doi:10.3390/plants14060871)

Figure S1. GO enrichment analysis

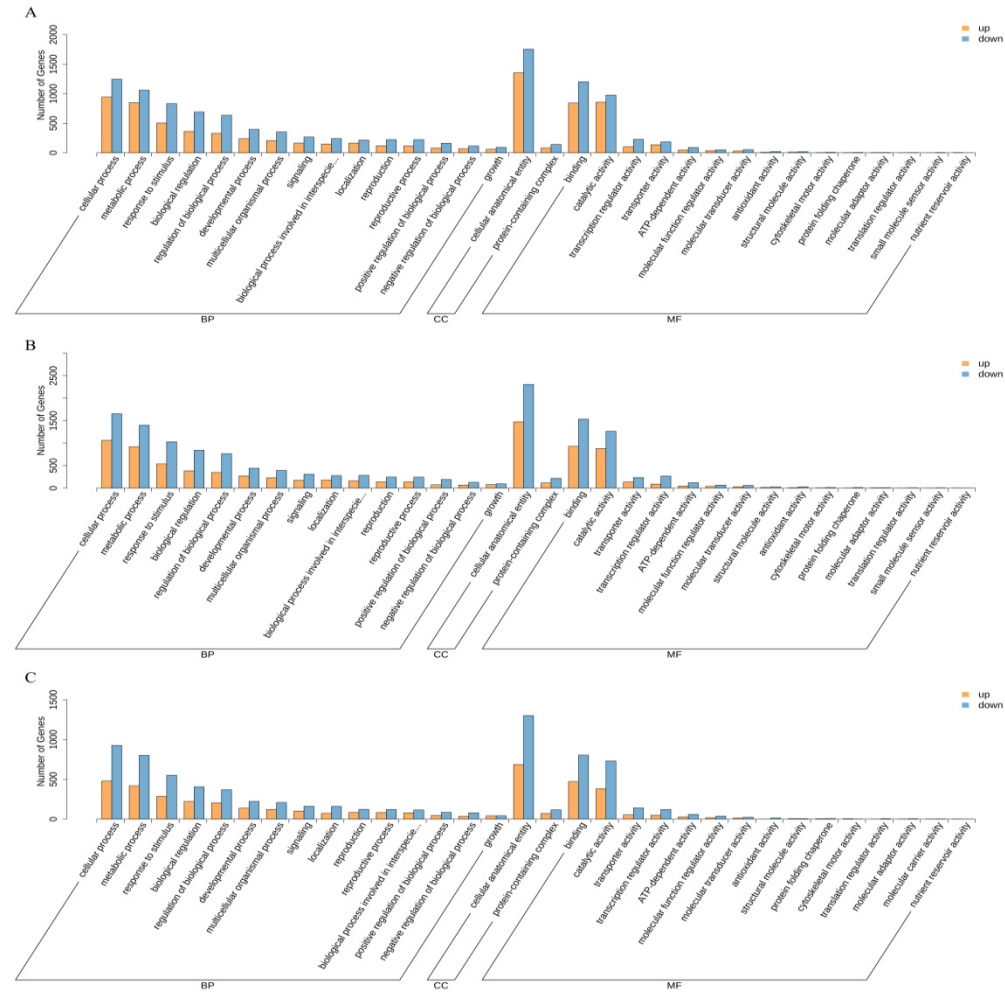

Figure S2. KEGG enrichment analysis

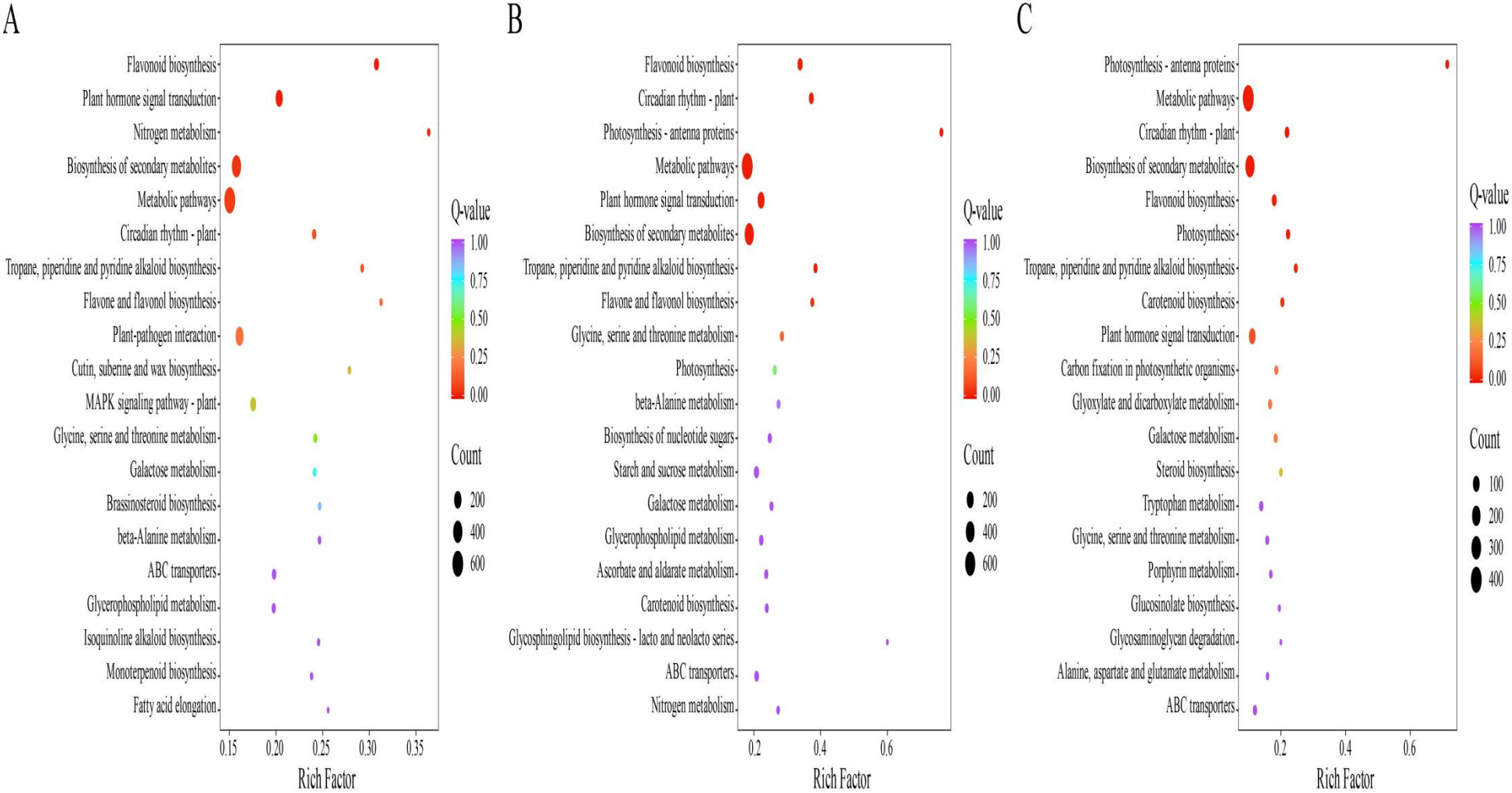

Note: A:S2-vs-S1 B:S3-vs-S1 C S3-vs-S2

Supplement: Supplementary file 1 [file plants-14-00871-s001.zip › Figure S1-S2. GO and KEGG Enrichment analysis in the transcriptome.pdf]

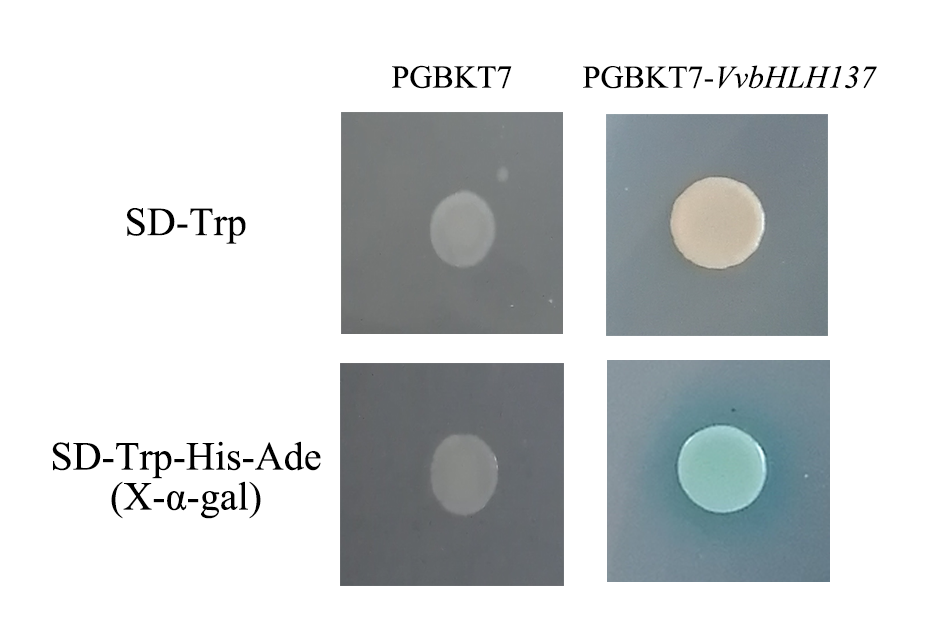

Supplement: Supplementary file 1 [file plants-14-00871-s001.zip › Figure S4. auto-activation of VvbHLH137.tif]
